# Supplementary material for: Preterm Infants’ Airway Microbiome: A Scoping Review of the Current Evidence
Source: Nutrients. 2024 Feb 6;16(4):465. doi: 10.3390/nu16040465 (PMC10891673; doi:10.3390/nu16040465)
Supplement: Supplementary file 1 [file nutrients-16-00465-s001.zip › nutrients-2789748-supplementary.pdf]

Ovid MEDLINE(R) ALL <1946 to November 22, 2023>

1 exp Infant, Premature/ or exp Infant, Premature, Diseases/ or Premature Birth/ or exp Infant, Low Birth Weight/ 135708

2 (((pre-matur* or premature* or pre-term* or preterm* or underweight or lbw) and (neonat* or new*-born* or newborn* or infan* or child* or baby or babies or birth or NICU*)) or low-birth-weight or low-birthweight or vlbw).ab,bt,kf. 148636

3 1 or 2 205181

4 exp Microbiota/ 78611

5 exp Respiratory System/ 542606

6 exp Mouth/ 321744

7 5 or 6 849753

8 4 and 7 3496

9 ((Lung* or pulmon* or mouth* or buccal or throat* or airway* or air-way* or trache* or nasal* or nose* or rhino* or bronch* or laryn* or pharyn* or respirat*) adj5 (microb* or micro-biot* or micro-biom* or microorganism* or micro-organism* or microflora or flora* or bacteri*)).ab,kf,ti. 32441

10 8 or 9 34356

11 3 and 10 326

Embase (Embase.com) session results (27 Nov 2023)

Results 511

(('pre matur*':ti,ab,kw OR premature*:ti,ab,kw OR 'pre term*':ti,ab,kw OR preterm*:ti,ab,kw OR underweight:ti,ab,kw OR lbw:ti,ab,kw) AND (neonat*:ti,ab,kw OR 'new* born*':ti,ab,kw OR newborn*:ti,ab,kw OR infan*:ti,ab,kw OR child*:ti,ab,kw OR baby:ti,ab,kw OR babies:ti,ab,kw OR birth*:ti,ab,kw OR nicu*:ti,ab,kw) OR 'low birth weight':ti,ab,kw OR 'low birthweight':ti,ab,kw OR vlbw:ti,ab,kw OR 'prematurity'/exp OR 'low birth weight'/exp) AND ('mouth flora'/exp OR 'lung microbiota'/exp OR (((lung* OR pulmon* OR mouth* OR buccal OR throat* OR airway* OR 'air way*' OR trache* OR nasal* OR nose* OR rhino* OR bronch* OR laryn* OR pharyn* OR respirat*) NEAR/5 (microb* OR 'micro biot*' OR 'micro biom*' OR microorganism* OR 'micro organism*' OR microflora OR flora* OR bacteri*)):ti,ab,kw)) 511

Web of science, 27/11/2023

159 record

((Lung* or pulmon* or mouth* or buccal or throat* or airway* or air-way* or trache* or nasal* or nose* or rhino* or bronch* or laryn* or pharyn* or respirat*) near/5 (microb* or micro-biot* or micro-biom* or microorganism* or micro-organism* or microflora or flora* or bacteri*)) (Topic) and (((pre-matur* OR premature* OR pre-term* OR preterm* OR underweight OR lbw) AND (neonat* OR new*-born* OR newborn* OR infan* OR child* OR baby or babies OR birth OR NICU*)) OR low-birth-weight OR low-

birthweight OR vlbw) (Topic) and Preprint Citation Index (Exclude – Database) and MEDLINE® (Exclude – Database)

Cinahl via Ebsco Host 27/11/2023

#	Query	Results
S11	S3 AND S10	127
S10	S8 OR S9	6,157
S9	TI (((Lung* or pulmon* or mouth* or buccal or throat* or airway* or air-way* or trache* or nasal* or nose* or rhino* or bronch* or laryn* or pharyn* or respirat*) n4 (microb* or micro-biot* or micro-biom* or microorganism* or micro-organism* or microflora or flora* or bacteri*))) OR AB (((Lung* or pulmon* or mouth* or buccal or oral or oro* or throat* or airway* or air-way* or trache* or nasal* or nose* or rhino* or bronch* or laryn* or pharyn* or respirat*) n4 (microb* or micro-biot* or micro-biom* or microorganism* or micro-organism* or microflora or flora* or bacteri*)))	6,082
S8	S4 AND S7	252
S7	S5 OR S6	78,461
S6	(MH "Mouth+")	20,522
S5	(MH "Respiratory System+")	60,004
S4	(MH "Microbiota")	3,113
S3	S1 OR S2	74,379
S2	TI ((((pre-matur* OR premature* OR pre-term* OR preterm* OR underweight OR lbw) AND (neonat* OR new*-born* OR newborn* OR infan* OR child* OR baby or babies OR birth* OR NICU*)) OR low-birth-weight OR low-birthweight OR vlbw)) OR AB ((((pre-matur* OR premature* OR pre-term* OR preterm* OR underweight OR lbw) AND (neonat* OR new*-born* OR newborn* OR infan* OR child* OR baby or babies OR birth* OR NICU*)) OR low-birth-weight OR low-birthweight OR vlbw))	58,109
S1	(MH "Infant, Premature+") OR (MH "Infant, Premature, Diseases+") OR (MH "Infant, Low Birth Weight") or (MH "Childbirth, Premature")	49,863
